# Draft Genome Sequence of the Phytopathogenic Fungus *Fusarium euwallaceae*, the Causal Agent of *Fusarium* Dieback

**DOI:** 10.1128/genomeA.00881-17

**Published:** 2017-08-31

**Authors:** Enrique Ibarra-Laclette, Diana Sánchez-Rangel, Eric Hernández-Domínguez, Claudia-Anahí Pérez-Torres, Randy Ortiz-Castro, Emanuel Villafán, Alexandro Alonso-Sánchez, Benjamín Rodríguez-Haas, Abel López-Buenfil, Clemente García-Avila, José-Abrahán Ramírez-Pool

**Affiliations:** a Red de Estudios Moleculares Avanzados, Instituto de Ecología AC, Xalapa, Veracruz, Mexico; b Cátedra CONACYT en el Instituto de Ecología AC, Xalapa, Veracruz, Mexico; c Servicio Nacional de Sanidad, Inocuidad y Calidad Agroalimentaria, Unidad Integral de Diagnóstico, Servicios y Constatación, Tecámac, Estado de México, Mexico

## Abstract

Here, we report the genome of *Fusarium euwallaceae* strain HFEW-16-IV-019, an isolate obtained from Kuroshio shot hole borer (a *Euwallacea* sp.). These beetles were collected in Tijuana, Mexico, from elm trees showing typical symptoms of *Fusarium* dieback. The final assembly consists of 287 scaffolds spanning 48,274,071 bp and 13,777 genes.

## GENOME ANNOUNCEMENT

*Fusarium euwallaceae* belongs to the ambrosia *Fusarium* clade (1). Species within this clade form symbiotic relationships with ambrosia beetles of the genus *Euwallacea* (Coleoptera: Curculionidae: Scolytinae) (2). An obligate symbiosis exists between *F. euwallaceae* and its *Euwallacea* sp. beetle host (3), and together they cause *Fusarium* dieback, which severely threatens natural forests, landscape trees, and avocado orchards (2–5). The Kuroshio shot hole borer (a *Euwallacea* sp.), an exotic ambrosia beetle native to Asia (1), is established in landscapes and forests in southern California in the United States (6) and was detected recently in Tijuana, Mexico (7).

The fungus was grown in potato dextrose broth (PDB) medium (29 ± 2°C, 200 rpm), and after 14 days, mycelia were harvested by filtration and cryogenically pulverized. Genomic DNA was isolated from 500 mg of pulverized tissue according to a previously described protocol (8).

Two DNA libraries were constructed using the Nextera XT DNA kit (Illumina). The libraries were quantified with a Qubit version 2.0 fluorometer (Thermo Fisher Scientific), and their quality was evaluated on a 2100 Bioanalyzer instrument (Agilent Technologies) using a 7500 DNA kit. Both libraries were sequenced on a MiSeq sequencer (Illumina) using a MiSeq version 2.0 reagent kit (300 cycles). Before assembly, paired-end reads (12,294,902) were filtered (see https://github.com/Czh3/NGSTools/blob/master/qualityControl.py) to obtain high-quality reads that were merged and adapter trimmed with SeqPrep (see https://github.com/jstjohn/SeqPrep). Over 11.2 million merged or paired-end reads were *de novo* assembled using Newbler version 3.0. Scaffolds were generated using the reference-based scaffolder MeDuSa (9) with the *Fusarium solani* genome as a guide for alignment. The assembly resulted in 287 scaffolds (788 contigs) totaling 48,274,071 bp (*N*_50_, 1,349,055 bp; largest scaffold, 4,967,455 bp; ~62.3× coverage). The genome assembly comprises a size comparable to that of other published genomes of *Fusarium* species (e.g., *F. fujikuroi*, 43.83 Mb [10]; *F. graminearum*, 36.44 Mb [11]; *F. oxysporum*, 61.35 Mb [12]; *F. verticillioides*, 41.77 Mb [11, 12] and *F. solani*, 51.21 Mb [13]).

Gene models were identified with the evidence-directed AUGUSTUS predictor (14), which was specially trained for *F. euwallaceae* using WebAUGUSTUS (15) and full coding sequences derived from a previously assembled transcriptome (unpublished data). AUGUSTUS gene models were improved/corrected using the Maker-P pipeline (16) and a database containing all of the proteins from the genomes of *F. fujikuroi*, *F. graminearum*, *F. oxysporum*, *F. verticillioides*, and *F. solani* (latest versions downloaded from the JGI/MycoCosm portal [see http://genome.jgi.doe.gov/programs/fungi/index.jsf]). The total number of genes predicted in the *F. euwallaceae* genome was 13,777, which is similar to the number of genes reported for *F. graminearum*, *F. fujikuroi*, *F. solani*, and *F. verticillioides* (13,322, 14,813, 15,705, and 15,869, respectively) and slightly lower than that of *F. oxysporum* (20,925). The genome completeness was assessed using BUSCO (17), which estimated the genome sequence to be 98.4% complete based on the presence of conserved orthologous gene sets specific to *Ascomycota* fungi.

The genomic data reported here will be useful to deepen our understanding of *Fusarium* dieback disease. Our preliminary analyses suggest that the *F. euwallaceae* genome encodes proteins homologous to those involved in the biosynthesis of polyketide-derived mycotoxins. Undoubtedly, *F. euwallaceae* can potentially produce an arsenal of toxins and virulence factors.

### Accession number(s).

This whole-genome shotgun project has been deposited at DDBJ/ENA/GenBank under the accession no. NHTE00000000. The version described in this paper is the second version, NHTE02000000.
